# Impactful factors and research design in CRISPR-edited stem cell research from top 10 highly cited articles

**DOI:** 10.1186/s13287-021-02471-x

**Published:** 2021-07-18

**Authors:** Michael Anekson Widjaya, Shin-Da Lee, Yuh-Shan Ho

**Affiliations:** 1 Department of Biotechnology, College of Health Science, Asia University, 500 Lioufeng Road, Wufeng, Taichung, 41354 Taiwan; 2 Department of Physical Therapy, China Medical University, Taichung, Taiwan; 3 Department of Physical Therapy, Asia University, No. 500, Lioufeng Road, Wufeng, Taichung, 41354 Taiwan; 4 School of Rehabilitation Medicine, Weifang Medical University, Shandong, China; 5 Trend Research Centre, Asia University, No. 500, Lioufeng Road, 41354 Wufeng, Taichung, Taiwan

**Keywords:** Citation analysis, Web of Science Core Collection, Stem cell, CRISPR/Cas9

## Abstract

Our objective in this review was to determine (1) impactful research articles about CRISPR-edited stem cells, (2) factors that affected CRISPR method performance in stem cell, and (3) research design related to CRISPR-edited stem cells. Screening research papers of related topic was carried out by using the Science Citation Index Expanded (SCIE) database of the Clarivate Analytics Web of Science Core Collection updated. We screened impactful CRISPR/Cas9-edited stem cells based on total citation until 2020. The result showed the title “RNA-guided human genome engineering via Cas9” was the highest citation in stem cell research using the CRISPR method with total citation 4789 from Web of Science Core Collection until 2020. It became the most influenced paper because this was the first research using CRISPR method for modifying human cells. On the other hand, cell type, CRISPR/Cas9 delivery, and gene target affected CRISPR/Cas9 performance in stem cells. The more complex the cell structure, the more difficult for CRISPR/Cas9 to mutate the host cells. This problem could be solved by modifying the CRISPR/Cas9 delivery by liposome and SaCas9 modification. Another way was using ribonucleoprotein (RNP) as a delivery method. Then, double gene target was more difficult to execute than single gene target. Although it is difficult, CRISPR/Cas9 had the capability to target any genome region from promoter until intron. Research design used a combination of dry lab and wet lab. The dry lab is usually used for sequence analysis and gRNA design. The wet lab which consisted of in vitro and in vivo was used for gene characterization. In particular, colony selection, DNA analysis, and sequencing were important parts for in vitro research design, while DNA analysis and sequencing were crucial parts for in vivo research design. We hoped these findings could give researchers, investor, and students a guideline to conduct CRISPR-edited stem cells in the future.

**Supplementary Information:**

The online version contains supplementary material available at 10.1186/s13287-021-02471-x.

## Background

Stem cells could be differentiated in many specific cell type [1] through modifying molecular signal [2]. Stem cells occurred in any organism from plants to humans [3] which could be applied to humans as a medical method [4]. They did it by replacing injured or abnormal human cell with the stem cell [5] while the original abnormal one would be removed [6]. However, stem cell had its limitation [7]. We needed to know the moment that the cell was in the stem cell phase because when the cell became adult stem cells, it would be difficult to be applied for the human medication [4]. Stem cell had a degree for specialization potential [7] which was totipotent, pluripotent, and multipotent [8]. Totipotent stem cell could specialize into anything but multipotent had the most limitation in the specialization cell process [9]. Another multipotent limitation was they sometimes could not integrate with cell’s recipient. Their mechanism had not yet been understood [7] which caused manipulating their differentiations not effective sometimes [6]. In addition, there was still only few evidence that stem cell transplantation worked especially in neuron damage patients [6].

**Fig. 1:**
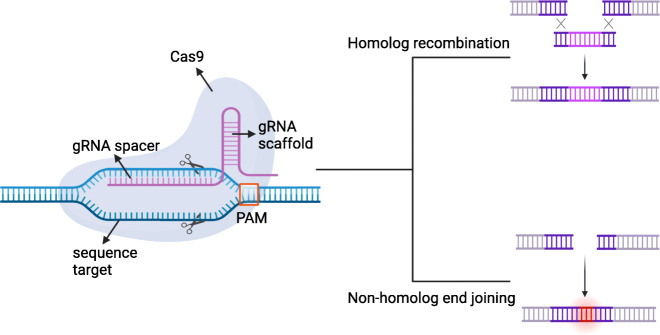
CRISPR basic mechanism. CRISPR/Cas9 consists of cas9 and gRNA. Cas9 will cleave the sequence target directed by gRNA. gRNA composition is based on 3 components: PAM, gRNA scaffold, and gRNA spacer. PAM function is to initially recognize the sequence target to cut. gRNA scaffold function supports Cas9 binding. gRNA spacer role is to attach the sequence target for cleavage. Cleavage impact is homolog recombination and non-homolog end joining. Pros are it is easy to design and more efficient compared to other methods. Cons are off-target effect and not effective in every cell type

However, the mentioned limitations could be solved thanks to the CRISPR (clustered regularly interspaced short palindromic repeats) method, especially if we wanted to know more about molecular biology in stem cells [10]. CRISPR is a genome editing method that was based on the prokaryote immune system [11]. In order to use CRISPR, scientists extracted CRISPR components such as Cas9 and sgRNA from bacteria [12]. The basic CRISPR mechanism was that the sgRNA guided the Cas9 protein to cleave the desired sequence which caused homolog direct recombination (HDR) or non-homolog end joining (NHEJ) (Fig. 1). Actually, genome editing in stem cell was common in order to know stem cell mechanism but it often made stem cell stressful [12]. Recent researches showed that CRISPR could overcome that problem because its mechanism was faster and more specific compared to another genome editing method [11]. Therefore, CRISPR could be the answer to a lot of questions in stem cell research in the present and future [13].

To confirm that opinion, we did a review using screening method that had been done in different subjects [14]. Through this method, our review analysis did not only show how many journals had been published in this subject but also showed the trending topic and its potential in the future [15]. The result led to determine (1) impactful research articles about CRISPR-edited stem cells, (2) factors that affected CRISPR method performance in stem cell, and (3) research design related to CRISPR-edited stem cells. The review analysis about CRISPR development for stem cell had not been mentioned in stem cell research before [16]. Also, this research was important for scientists to trace the possibility that stem cells could be integrated with CRISPR especially as the CRISPR-based biotechnology industry was developing [17]. In order to support that notion, we also provided research design about CRISPR-edited stem cell research to give insight for scientist, investor, and students who wanted to learn about this subject for future application. That was the reason we decided to write CRISPR for stem cell development review, which was to provide a guide and find out how much CRISPR affected stem cell research.

On the other hand, the database that we used in this research was the Science Citation Index Expanded (SCIE) because it had been used for stem cell research bibliometric analysis before [16] and covered many journals publications more than decades [18]. During the writing process, we excluded review and any other kind of papers. Thus, we only focused on research articles. We made the selection from published papers from 2013 until 2020. This subject was relatively new since it had been found [19]. Therefore, the oldest CRISPR-related research papers were 2013.

## Screening methods

Web of Science Core Collection was the most effective site for trend studies because it existed since 1900 [20, 21]. The data in this study was found through one of the Web of Science Core Collection branch called the Science Citation Index Expanded (SCIE) database of the Clarivate Analytics (data last updated on May 25, 2021).

After our introductory study, the keywords such as “stem cell,” “stem cells,” and “meristem cells” as well as “CRCRISPRSPRSPR,” “CRISPRmediated,” “ICRISPR,” “CRISPRed,” “CRISPRing,” “CRISPRs,” “CRISPRa,” “CRISPRi,” “CRISPRai,” and “CRISPR” were used in the topic discipline, including paper title, abstract, author keywords, and *KeyWords Plus*, in the Web of Science Core Collection for the year issue between 1900 and 2020. *KeyWords Plus* gave supporting information that was filtered from the titles of the articles referenced by scientists in their references and footnotes in Clarivate Analytics database and considerably build up author-keyword indexing and title-word [22]. This result produced 2663 documents as stem cell and CRISPR publications. The oldest document was found in 2013 which caused the discussion to be started from 2013. Those documents were only set up by *KeyWords Plus* and were potentially uncorrelated to the “stem cell and CRISPR” [14]. It meant we need another confirmation. Ho’s team firstly suggested filtering documents based on the “frontpage” [15, 23, 24]. They are the title, abstract, and author keywords in the “front page” part; unrelated publications might be reduced by this filter for analysis [15]. Finally, 1888 documents (71% of 2663 documents) were categorized as stem cell and CRISPR research publications. The full record of SCI-EXPANDED and the citation frequency in each year for each document were collected in Microsoft Excel 2016 format and evaluated. The additional modification was performed manually [23, 25]. Only 1405 articles were further analyzed.

There are four citation indicators we used to obtain citation information which was received by the publications:

*C*_0_: The citations frequency from the Web of Science Core Collection in publication year [26]

*C*_year_: The citations frequency from the Web of Science Core Collection in the most recent year

*C*_2020_: The number of citations in 2020 [27]

*TC*_year_: The total citations frequency from the Web of Science Core Collection since publication to the end of the most recent year. In this study, this is 2020 (*TC*_2020_) [28].

*CPP*_year_: Citations per publication (*CPP*_2020_ = *TC*_2020_/*TP*); *TP* is total number of publications [27].

## Result and discussion

**Fig. 2:**
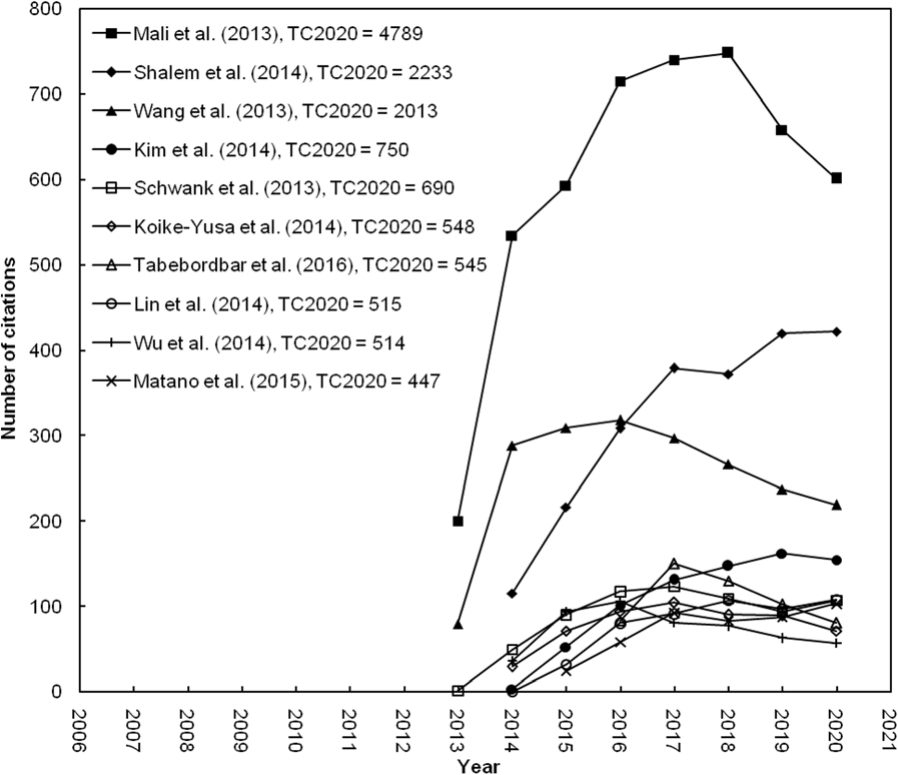
The citation histories of the top 10 highly cited articles with *TC*_2020_ ≥ 447

Total reference information from the Web of Science Core Collection was updated weekly. In order to improve the bibliometric study, the total citations frequency from Web of Science Core Collection since publication to the end of 2020 (*TC*_2020_) was applied to reduce the bias using data from Web of Science directly. The benefit of *TC*_2020_ value was stable and consistent compared with the citation index from the Web of Science Core Collection [15]. The citation histories of the top ten most frequently cited stem cell and CRISPR articles (*TC*_2020_ ≥ 477) are found in Fig. 2. Three of the top ten articles were published in 2013, while the last one was published in 2016. The article by Mali et al. [19] ranked top in *C*_0_, *C*_2020_, and *TC*_2020_. Articles by Shalem et al. [29, 30] also had high citations after their publications. It was pointed out that highly cited publications do not always bring high impact or visibility after it was published [31]. The amount of citation received in the year 2020 (*C*_2020_) and in the publication year (*C*_0_) might provide supporting content for readers to understand the highly cited article impact today versus its immediate effect post publication [27]. The 1405 articles in stem cell and CRISPR research ranked differently if sorted by *TC*_2020_, *C*_2020_ than sorted by *C*_0_. A total of 274 articles (20% of 1405 articles) had no citation in the most recent year (*C*_2020_ = 0) and 693 (49%) articles had no citation in the initial publication year (*C*_0_ = 0). Furthermore, among the top 100 *C*_0_ articles, 45% and 47% of the articles were among the top 100 *TC*_2020_ and *C*_2020_ articles, respectively. In recent years, high-impact research journals in the most recent year in a Web of Science category [27, 31, 32] and a research topic [14, 15, 33] were evaluated by using a citation indicator, *C*_year_. Seven of the top ten highly cited articles with *TC*_2020_ ≥ 447 were found to be the top ten high-impact articles in 2020 with *C*_2020_ ≥ 103.

## The impactful articles in stem cell and CRISPR research

**Fig. 3:**
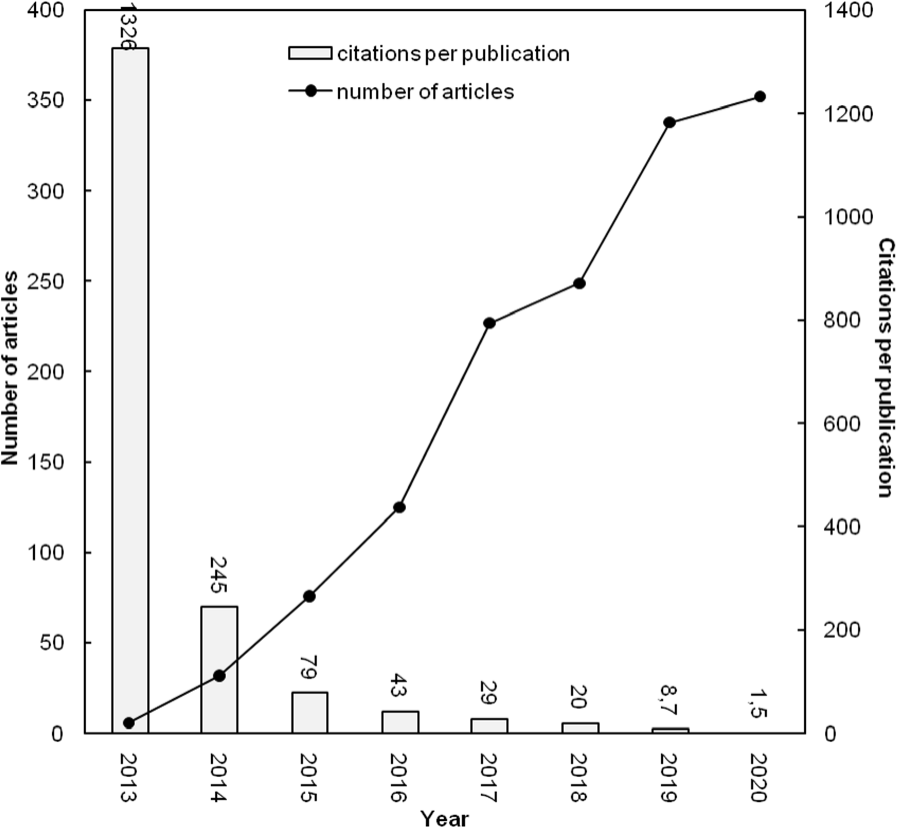
Number of stem cell and CRISPR articles and citations per publication by year

We measured impactful articles based on total citation until 2020 (Fig. 3). From the analysis, it showed this topic of stem cell and CRISPR research was found from 2013 and it increased every year. It came from a research regarding microbiology component called Cas9 which could be applied in human engineering [19]. The first research who had been done by team Mali [19] showed CRISPR/Cas9 method was better than TALEN (transcription activator-like effector nucleases) method because homolog recombination (HR) using CRISPR/Cas9 percentage was higher than the TALEN method. Not only for the HR factor, the CRISPR/Cas9 method was also faster than TALEN method. As a consequence, the research had *TC*_2020_ of 4789 and *C*_2020_ of 601 (Table 1) and the research trend increased gradually because CRISPR/Cas9 performance was better than conventional genomic editing for particular, TALEN (Fig. 2).

**Table 1:** The top ten most frequently cited articles

Rank (*TC*_2020_)	Rank (*C*_2020_)	Article title	Country	Reference
1 (4789)	1 (601)	RNA-guided human genome engineering via Cas9	USA	Mali et al. [19]
2 (2233)	2 (422)	Genome-scale CRISPR-Cas9 knockout screening in human cells	USA	Shalem et al. [30]
3 (2013)	3 (219)	One-step generation of mice carrying mutations in multiple genes by CRISPR/Cas-mediated genome engineering	USA	Wang et al. [29]
4 (750)	5 (154)	Highly efficient RNA-guided genome editing in human cells via delivery of purified Cas9 ribonucleoproteins	South Korea	Kim et al. [12]
5 (690)	9 (107)	Functional repair of CFTR by CRISPR/Cas9 in intestinal stem cell organoids of cystic fibrosis patients	Netherlands	Schwank et al. [34]
6 (548)	16 (71)	Genome-wide recessive genetic screening in mammalian cells with a lentiviral CRISPR-guide RNA library	UK	Koike-Yusa et al. [35]
7 (545)	14 (80)	In vivo gene editing in dystrophic mouse muscle and muscle stem cells	USA	Tabebordbar et al. [36]
8 (515)	8 (108)	Enhanced homology-directed human genome engineering by controlled timing of CRISPR/Cas9 delivery	USA	Lin et al. [37]
9 (514)	25 (57)	Genome-wide binding of the CRISPR endonuclease Cas9 in mammalian cells	USA	Wu et al. [38]
10 (447)	10 (103)	Modeling colorectal cancer using CRISPR-Cas9-mediated engineering of human intestinal organoids	Japan	Matano et al. [39]

Various genes were explored more in vitro by team Shalem because Mali et al.’s research only explored *AAVS1* as a gene target [19, 30]. Their research became number two most cited articles because of that contribution. Then, the medicine application was applied by team Schwank [34]. They applied the knockout-knockin mutation to recover cystic fibrosis cells which was success although the percentage was low (Additional file 2). From 2013 to 2015, they did experiment in vitro more often to explore various gene target, delivery method, and cell types. A research even started an in vivo experiment which had been done by team Wang [29]. They used zygote as the host cells for CRISPR/Cas9 genome editing and targeted two genes at the same time. This research design made their research became the number three most cited articles with *TC*_2020_ of 219 because this was the first time in vivo-related research occurred (Table 1). Kim et al.’s research explored more in gene delivery using Cas9 extraction from *Escherichia coli* and ribonucleoprotein (RNP) delivery caused the gene mutation that occurred to be more effective [12]. This finding led them into fourth rank most cited articles. Rank sixth impactful articles showed Koike-Yusa et al.’s research which was about genomic modification in embryonic stem cells (ESCs) (Additional file 1). Their result showed higher modification compared to Mali et al.’s research (Additional file 2). The eight ranked impactful article was Lin et al.’s research which was about cells receiving better CRISPR/Cas9 genomic materials if the cell time was at the M (mitotic) phase [37]. Another suggestion about CRISPR performance in stem cell was also discussed in Wu et al.’s research (rank 9th TC_2020_). They proposed that seed sequence which was close to PAM (proto adjacent motif) region must be a complement to the target sequence in order to increase genome editing specificity [38]. The least rank was Matano et al.’s research about CRISPR performance in intestinal organoid for designing a cancer model [39]. Their result was consistent with Schwank’s result which was low percentage mutation (Additional file 2).

Later, after 2016, a research about in vivo research was explored again but by a different team, Tabebordbar et al.’s research. The research explored mice which had dystrophic muscle. It showed that the dystrophic muscle with CRISPR/Cas9 treatment showed more effective recovery [36]. Interestingly, this research ranked seventh and surpassed Lin et al.’s research which was published 2 years earlier. Another medicine-themed research also entered rank four in this paper from Schwank research (Additional file 1). This phenomenon conveyed that medicine research could be dominant in CRISPR-edited stem cell in the future.

## Cell type, CRISPR/Cas9 delivery, and gene target affected the CRISPR/Cas9 editing performance

The ten most impactful articles showed cell type, CRISPR/Cas9 delivery, and gene target affected CRISPR/Cas9 performance as a genome editing tool in stem cells (Additional file 2). Those factors were important for stem cell scientist who wanted to conduct CRISPR-edited stem cells. Different cells showed different sensitivity against CRISPR/Cas9 delivery. Their success mutation efficiency was different among the other cells. CRISPR/Cas9 delivery consisted of three delivery types. There were plasmid, virus, and RNP. Gene target in this paper had various functions from marker, expressed protein until intron. In this part, we would discuss one by one from cell type until gene target role in this topic.

**Fig. 4:**
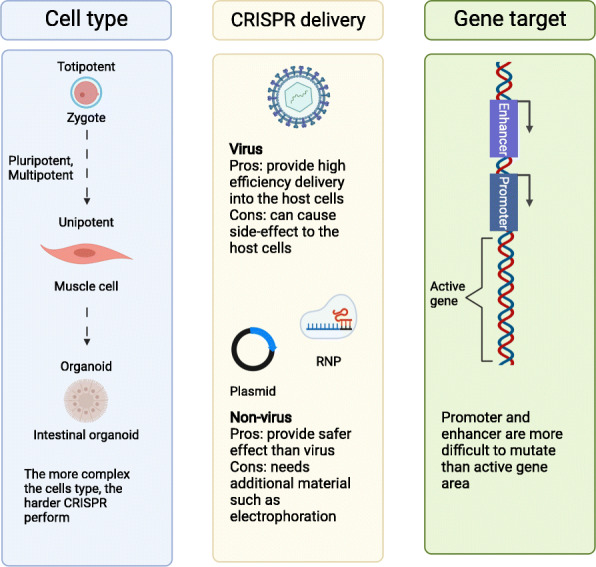
Cell type, delivery methods, and gene target affected CRISPR performance in stem cell

Cell type factors that influenced the CRISPR/Cas9 were complexity and health condition (Fig. 4). The more complex the cells, the more difficult it is for CRISPR/Cas9 to penetrate into the cells. Schwank et al.’s and Matano et al.’s research showed only 1.6% and 0.03% cell mutation respectively compared to other research which only used in vitro cells (above 3%) (Additional file 2). Furthermore, pluripotency would decrease in organoid level which would affect the CRISPR/Cas9 efficiency. For instance, mice zygote which pluripotency was still high and showed higher efficiency compare to another cells (> 90%). The pluripotency itself must be natural because induced pluripotency stem cells showed low efficiency (2–4%). Compared among “simple” cell level, the cancer cells were the easiest to receive the CRISPR/Cas9. Shalem et al.’s research showed over 90% cells got mutation compared to other cells which were below 72%. However, this obstacle related to cell complexity which could be solved by modified gene delivery. Tabebordbar which was an in vivo experiment showed better mutation efficiency compare to organoid research (3–18%:1.6%). Tabebordbar used different gene delivery which was unconventional compared to other papers. This part would be discussed in the next part.

Delivery method was a crucial factor when we wanted to make sure the CRISPR/Cas9 penetrated the host cells successfully. Research showed that plasmid delivery especially liposome type was more successful than viral delivery (> 50%:< 47%) (Additional file 2). Viral delivery was not consistent; sometimes it hit below 2%; and sometimes, it was higher than 90% (Additional file 2). However, if we compared with same host cells which were same cells type, liposome was better based on Mali et al.’s research (lentiviral) and Kim et al.’s research (liposome) comparison (13–38%:57–72%). Another delivery method, RNP method, showed good efficiency. The result was better compared to Mali et al.’s research (38–50%:10–25%) (Additional file 2). This finding showed RNP delivery could be explored more in the future beside liposome delivery. Another important finding was new Cas9 for executing the gene mutation. New Cas9 type, SaCas9, which was extracted from *Staphylococcus aureus*, showed high efficiency in vivo. Unfortunately, the research used viral vector which was not good compared liposome delivery. This might be the reason why the efficiency was lower than another in vivo research, Wang et al.’s research (Additional file 2). However, good efficiency for in vivo animal showed that it could be better for in vitro research. Also, SaCas9 required lesser sequences compare to Cas9 protein which caused more space for gRNA sequences inside the vector [40]. In the end, two findings which could be explored in the future were SaCas9 and RNP. Those components could improve the CRISPR/Cas9 efficiency for complex cells.

Research papers showed that CRISPR/Cas9 could hit any gene target from various functions and area. Various gene functions in particular secreted protein function (*GFP*), integrated function (*AAVS1*), and protein membrane (*CXCR4*) could be mutated by CRISPR/Cas9. Another research showed that any region from intron, enhancer, promoter, and exon could be edited by CRISPR/Cas9 [30]. However, active gene was easier to be edited compared to another region, for example, enhancer and promoter [38]. The easiest genes were *AAVS1* and *GFP* based on high percentage and ten papers’ research design. Usually, they would mutate those genes in order to confirm the CRISPR/Cas9 performance. This could be used as a reference for stem cell scientist when they tried to conduct CRISPR-edited stem cell for the first time. Double gene mutation was more difficult than single gene mutation. The percentage was lower than the single gene mutation (Additional file 2). Even when the research tried to do knockin, it would be more difficult. These obstacles needed to be solved in the future if the CRISPR/Cas9 application in stem cells experiment would be conducted more often.

## CRISPR-edited stem cell research design

**Fig. 5:**
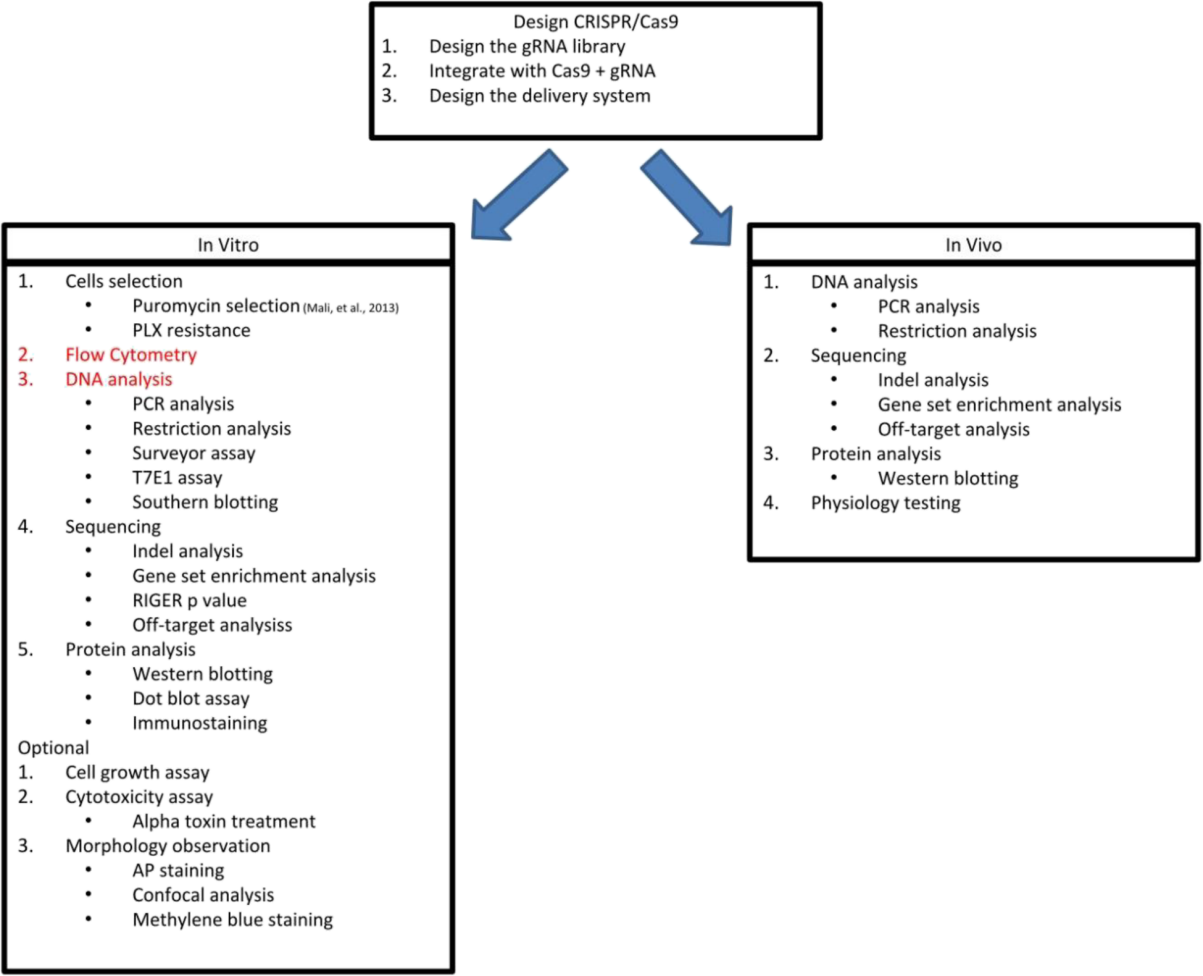
Research design in vitro and in vivo for CRISPR-edited stem cells was different. Red text means those experiments were not in order. Some research ignored one of those experiments

Stem cell research design was a combination of dry lab and wet lab. The dry lab was used for design of the gRNA library and sequencing the result for further analysis [12, 19, 29, 30, 34–39]. The first stage was called the design of the CRISPR/Cas9 in order to target the desired gene accurately (Fig. 5). In this phase, we designed a lot of gRNA sequence-based bioinformatics database like NCBI [30]. Another method was using inverse PCR to produce many gRNA target with unknown sequences [34]. gRNA sequences were selected based on mismatch prediction and mutation type. Those factors were calculated to predict the off-target chance. The lowest off-target score usually would be selected for vector integration with plasmid or virus. After the integration was done, it would be transfected into the stem cell target for enrichment. The cells were selected based on negative or positive screen which was part of in vitro experiment (Fig. 5).

In vitro experiment was started with cell mutation screening using various methods (Fig. 5). However, some experiments only did one of the experiment or both, depending on the research purpose. If the purpose did not need to pay attention to cells’ characteristics too much, we could select puromycin selection only. Several research did that because the gene did not express special characteristics beside GFP (green fluorescent protein) or puromycin resistance [19, 29, 30, 34, 35]. However, another research did additional selection which was PLX resistance because when the gene was knockout, the cells became resistant against BRAF protein kinase inhibitor vemuranefib (PLX) [30]. This selection was used to characterize further the cell profile which did not appear only based on puromycin selection. In an alternative way, if this experiment was not possible to be conducted, the next step was flow cytometry (Fig. 5). This could be used as another way or following step after cell selection [19, 30, 35]. A research paper did flow cytometry analysis directly without cell selection [37]. From this experiment, the cells were selected based on GFP expression as a sign CRISPR/Cas9 was transfected successfully. Another reason was used to confirm homolog recombination (HR) was successfully inserted into the cell chromosome. A marker was put into HR gene target usually [19]. Interestingly, a research did not test the step 1 and step 2 (Fig. 5). The research just did directly step 3 which was DNA analysis [12]. This was not a popular research design because only one impactful research paper did this experiment directly among ten CRISPR-edited stem cell research papers. This step was standard procedure in CRISPR/Cas9 topic because every papers would do this step after step 1 and step 2 [19, 29, 30, 34, 37]. Only one paper did not do this analysis [35]. Majority of the papers did this experiment because the purpose for DNA analysis was to make sure the gene target was cut out because of CRISPR/Cas9 action. The activity was measured by various methods. For instance, PCR analysis and southern blotting detected the DNA directly from the cell condition. RFLP method was detected by restriction enzyme treatment before the DNA was injected into gel electrophoresis. Surveyor assay and T7E1 assay detected mismatch in DNA directly but T7E1 had specific purpose for CRISPR/Cas9 DNA product detection. The next step was sequencing. After the band from DNA analysis was received by DNA isolation, it would be sequencing to track the mismatch or difference compare to control DNA. All of the papers did this experiment [12, 19, 29, 30, 34, 35, 37–39]. However, a paper did this without doing DNA analysis step [35]. This step was alternative if we did not want to do DNA analysis because sequencing could know everything directly, although confirmation is needed to be done like flow cytometry. From sequencing, we knew about mismatch profile, off-target accuracy, gene expression, or performance comparison between CRISPR/Cas9 and another genomic editing such as shRNA [30]. The final step was protein analysis to confirm the protein from mutant cells was expressed or not. Three researches did this experiment after sequencing [12, 29, 30], while the rest of the impactful articles did not confirm this. A total four papers confirmed protein expression because the gene target was a secreted protein type or wanted to confirm protein Cas9 durability [12, 29, 30, 39].

Optional step was done for specific purpose which meant not every paper did this step. This step particularly was applied to characterize the cell longevity under CRISPR/Cas9 treatment through cell growth assay [30]. Another purpose was to understand the toxic resistance through cytotoxicity assay because their gene target could improve that ability [35]. Characterization could also be done by staining observation to show the gene was expressed or not through alkaline phosphatase staining [12], confocal analysis [34], and methlyene blue staining [35].

In vivo research design was less complicated than in vitro experiment. Based on review analysis, only two papers did in vivo experiment [29, 36]. The analysis was almost same with in vitro experiment but it did not need colony selection (Fig. 5). Furthermore, physiology which could not be done in vitro was done exclusively for this experiment design by measuring the muscle force as an example [36]. On the other hand, in situ hybridization and immunostaining were done to detect mutated organoid integration into the mice for cancer purposes [39].

Overall, conducting CRISPR/Cas9-edited stem cells research required knowledge from the bioinformatic and molecular biology field. The first phase was dry lab to confirm gRNA, Cas9, and delivered vector design. The second phase could be in vitro or in vivo experiment, depending on the CRISPR/Cas9 performance. If the CRISPR/Cas9 performance was unknown, in vitro research should have done first, then moved to in vivo experiment. Both in vitro and in vivo experiment were almost the same except the colony selection part which was only done in the in vitro phase.

## Conclusion

Research in stem cells using CRISPR methodology was found in 2013 and it showed better performance compared to conventional genomic editing. This led to more research about understanding CRISPR/Cas9 performance in any condition from in vitro until in vivo. Those studies tested various cell type, CRISPR/Cas9 delivery, and gene target with different purposes, from basic science research until medicine. The result showed that the more complex the cell structure, the more difficult for CRISPR/Cas9 to change the genomic component. However, this obstacle could be solved by modified CRISPR/Cas9 delivery by liposome delivery and replaced Cas9 with SaCas9. Although the delivery system solved the problem, gene target also needed to pay attention in the future because double gene mutation was more difficult to be done than single gene mutation. Research design in this topic consisted of in vivo and in vitro studies. Most of them were similar from gRNA design until gene characterization. However, the difference was the in vivo study did not need colony selection. Instead, the research just did directly DNA analysis and sequencing mostly. We expected that the research design that we elaborated could help stem cell scientist to understand more about CRISPR/Cas9-edited stem cell research design pattern in order to make the CRISPR/Cas9 development in stem cell faster.

## Supplementary Information

**Additional file 1.** Top 10 highly cited articles overview. This data is about the research scheme from each study to give more insight about the experiment process.

**Additional file 2.** Overall top 10 stem cell using CRISPR method researches table. TC2020 = Total Citation until 2020.

## Data Availability

All data generated or analyzed during this study are included in this published article.

## Abbreviations

CRISPR: Clustered regularly interspaced short palindromic repeats
Cas9: CRISPR-associated protein 9
gRNA: Guide RNA
PAM: Proto adjacent motif
SCIE: Science Citation Index Expanded
TC: Total citations
TALEN: Transcription activator-like effector nucleases
HR: Homolog recombination
HDR: Homolog direct recombination
*AAVS1*: Adeno-Associated Virus Integration Site 1
RNP: Ribonucleoprotein
ESCs: Embryonic stem cells
M: Mitotic
NHEJ: Non-homolog end joining
SaCas9: *Staphylococcus aureus* CRISPR-associated protein 9
GFP: Green fluorescent protein
CXCR4: CXC-chemokine receptor 4
PLX: Vemuranefib
T7E1: Terminal 7 Endonuclease 1
RFLP: Restriction fragment length polymorphism
BRAF: B-Raf proto-oncogene
shRNA: Short hairpin RNA
*CFTR*: Cystic fibrosis transmembrane conductance regulator
