# Supplementary material for: Impactful factors and research design in CRISPR-edited stem cell research from top 10 highly cited articles
Source: Stem Cell Res Ther. 2021 Jul 18;12:411. doi: 10.1186/s13287-021-02471-x (PMC8286559; doi:10.1186/s13287-021-02471-x)
Supplement: Supplementary file 1 — Additional file 1. Top 10 highly cited articles overview. This data is about the research scheme from each study to give more insight about the experiment process. [file 13287_2021_2471_MOESM1_ESM.docx]

| TC_2020_ rank | First Author | Major research scheme |
| --- | --- | --- |
| 1 | Mali | 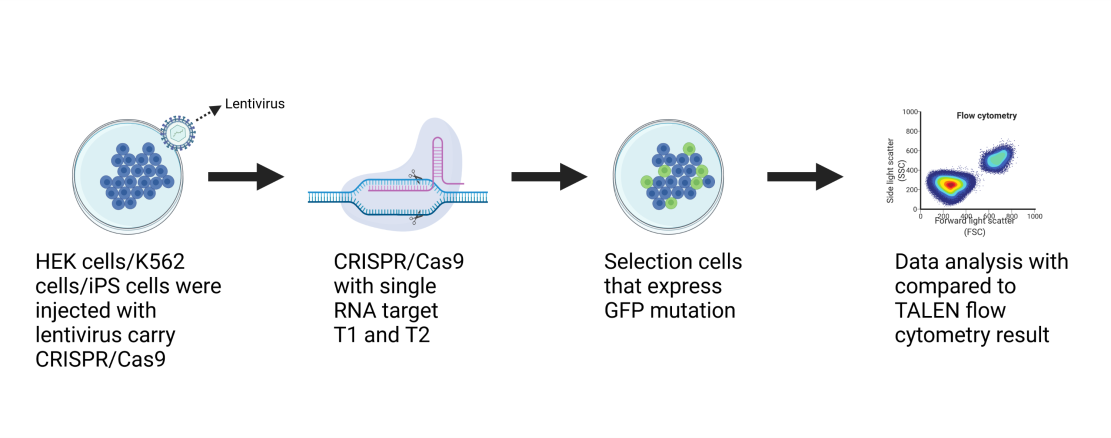 |
| 2 | Shalem | 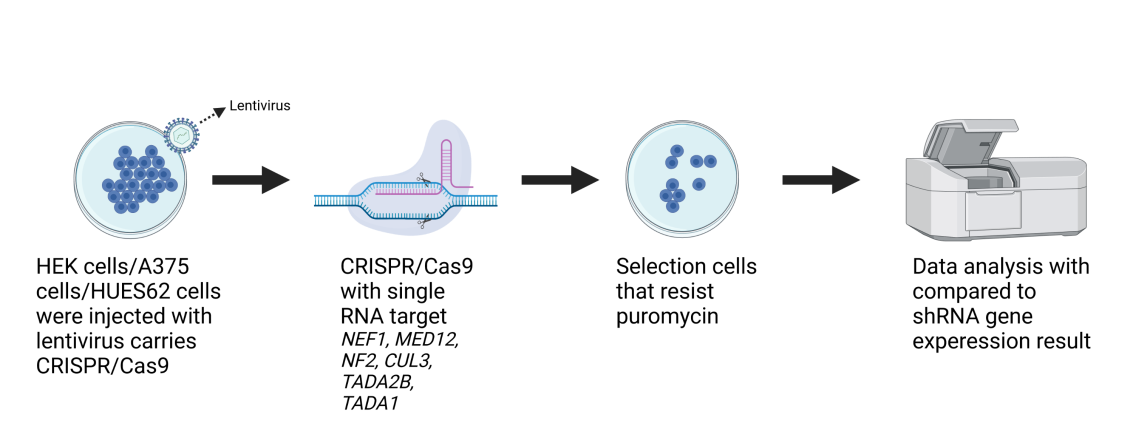 |
| 3 | Wang | 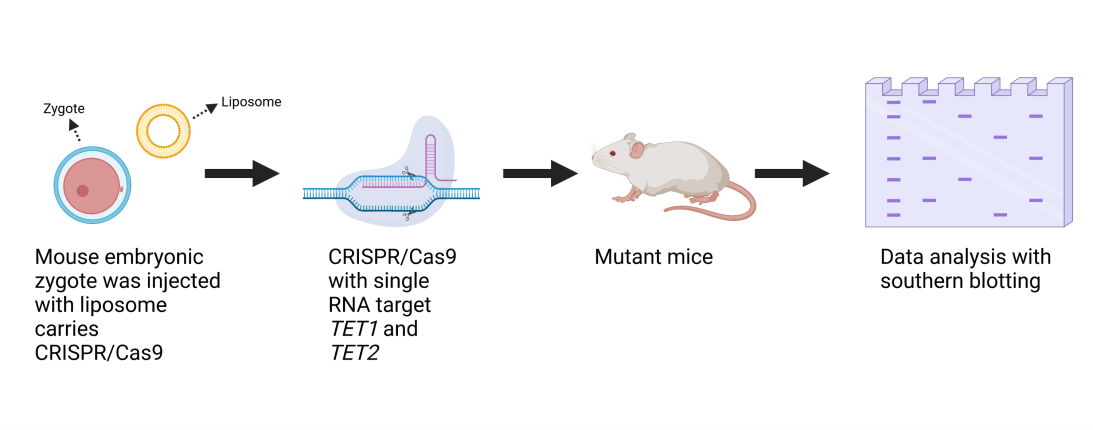 |
| 4 | Kim | 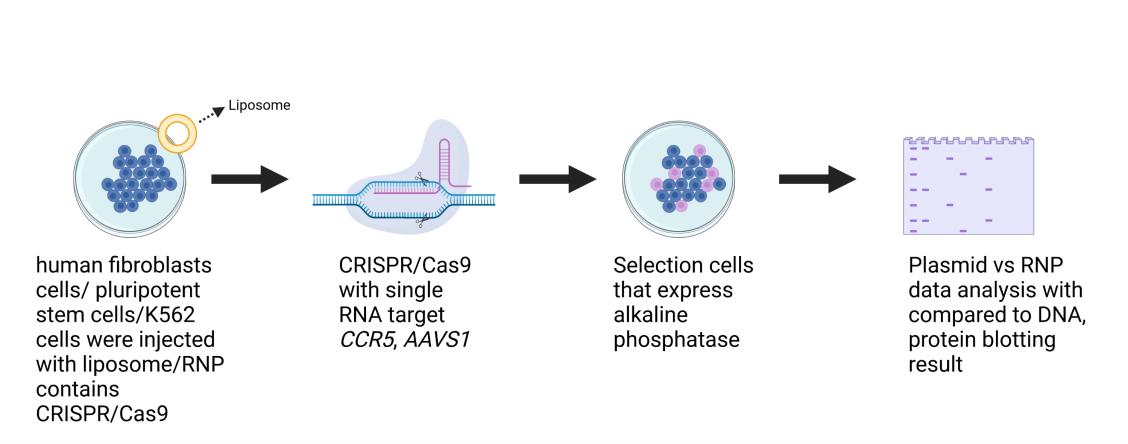 |
| 5 | Schwank | 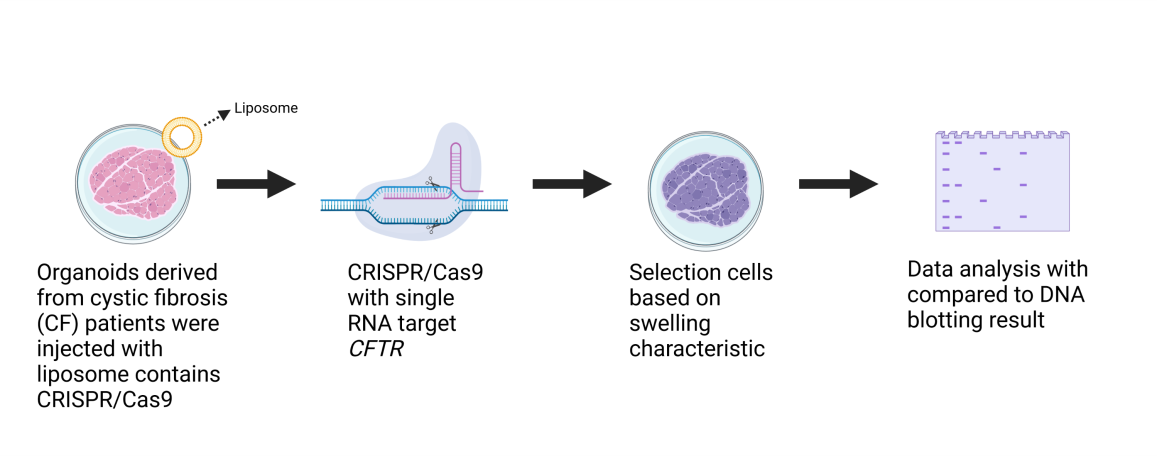 |
| 6 | Koike-Yusa | 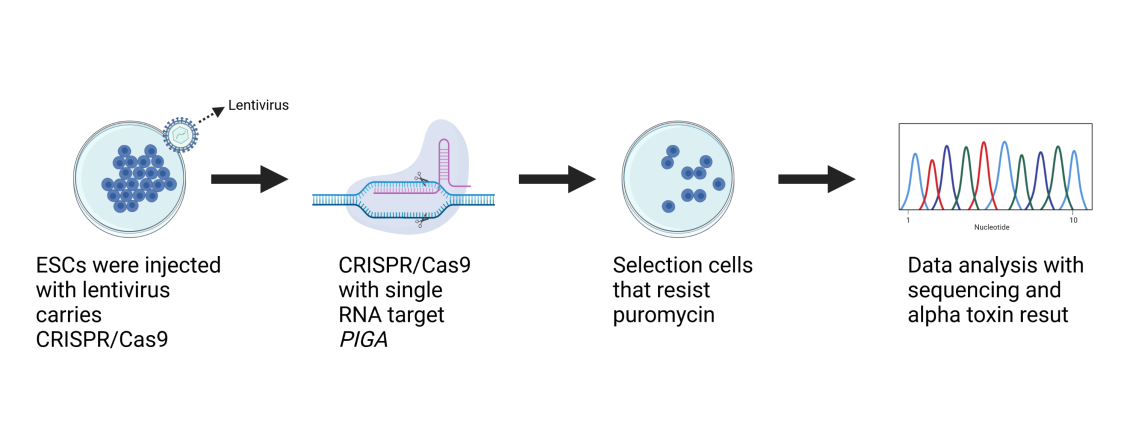 |
| 7 | Tabebordbar | 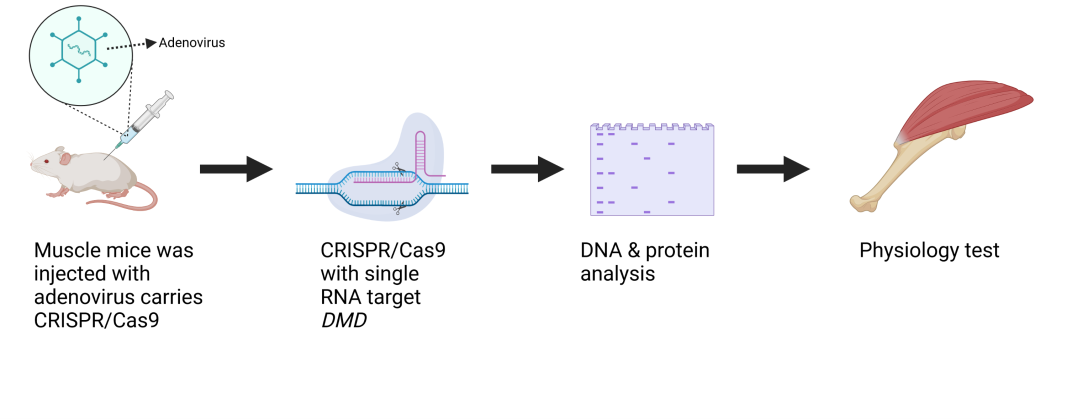 |
| 8 | Lin | 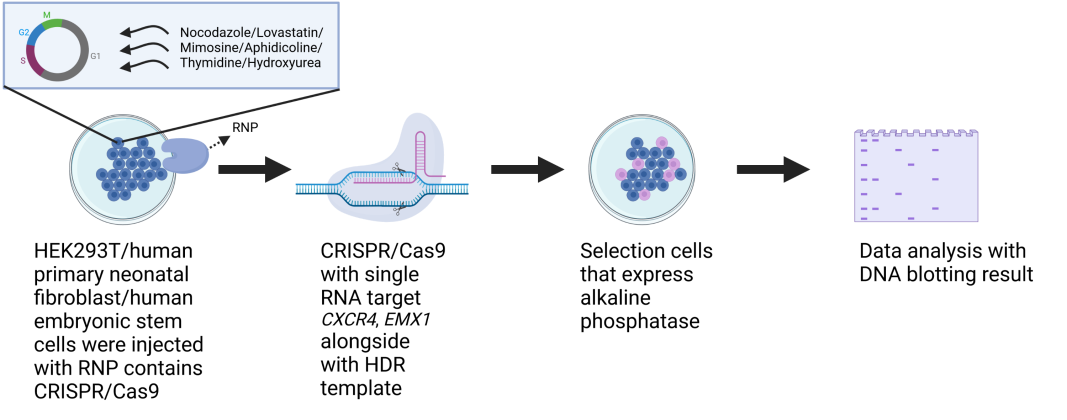 |
| 9 | Wu | 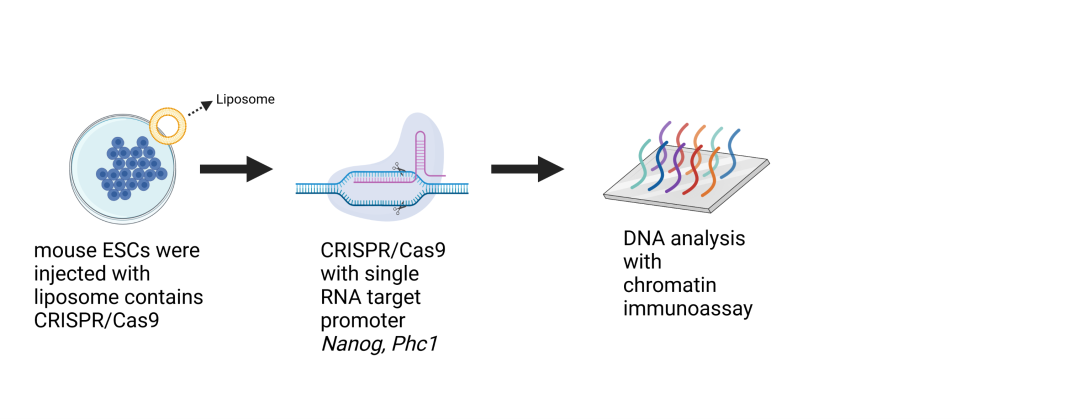 |
| 10 | Matano | 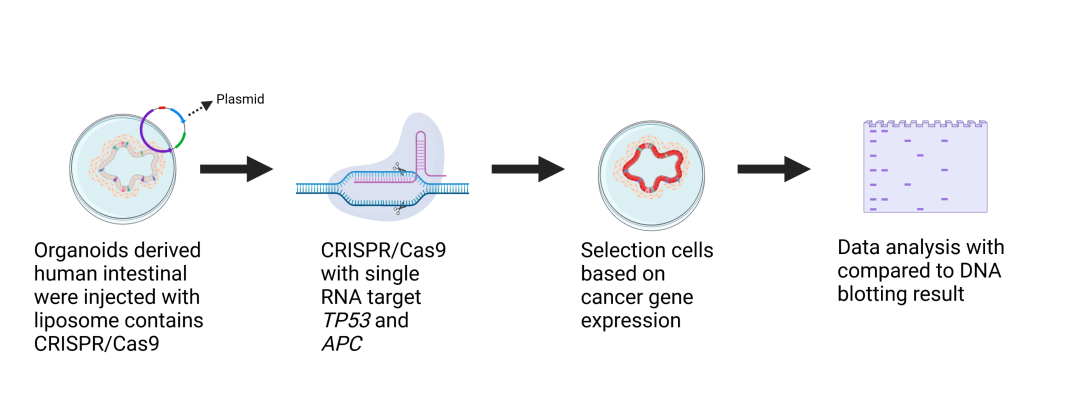 |
