# Supplementary material for: Impactful factors and research design in CRISPR-edited stem cell research from top 10 highly cited articles
Source: Stem Cell Res Ther. 2021 Jul 18;12:411. doi: 10.1186/s13287-021-02471-x (PMC8286559; doi:10.1186/s13287-021-02471-x)
Supplement: Supplementary file 2 — Additional file 2. Overall top 10 stem cell using CRISPR method researches table. TC2020 = Total Citation until 2020. [file 13287_2021_2471_MOESM2_ESM.docx]

| Research (Year) | Objective | Stem Cell Type | CRISPR type + Delivery system | Description |
| --- | --- | --- | --- | --- |
| Mali (2013) 1^st^ TC2020 | Testing CRISPR application in human stem cell | a. Human embryonic kidney (HEK) 293T cell  b. human chronic myelogenous leukemia K562 cells  c. PGP1  human induced pluripotent stem (iPS) cells | CRISPR-Cas9 with single gRNA.  (T1 and T2)  Lentivector | i. In vitro research  ii. Gene edit  *AAVS1* locus  a. 10-25% in 293T cells (embryonic kidney)  b. 13-38% in K562 cells (human chronic myelogenous leukemia)  c. 2-4% in induced pluripotent stem cells |
| Shalem (2014)  2^nd^ TC2020 | Knockoout human cells | a. HEK293T cell line  b. A375 melanoma cell  c. HUES62 | CRISPR-Cas9 with single gRNA knockout (GeCKO)  Lentivector | i. In vitro research  ii. Gene edit  *NF1, MED12*  a. >98% gene knockout  iii. *NF2*  a. *>*90% gene knockout  iv. *CUL3*, *TADA2B*, and *TADA1*  a. Cell survival rate increase |
| Wang (2013) 3^rd^ TC2020 | Generate mutation in mice genes | Mouse embryonic stem zygote | CRISPR-Cas9 with single gRNA  Liposome | i. In vivo research  ii. 80% mutation to gene *Tet1* and *Tet2*  iii. 13% mice receive double-gene targeting mutation |
| Kim (2014)  4^th^ TC2020 | Change human cells gene using purified Cas9 | a. human fibroblasts  b. human pluripotent  stem cells  c. K562 cells | CRISPR-Cas9 with single gRNA  RGEN ribonucleoproteins (RNPs)  Liposome | i. In vitro research  ii. Gene edit  *CCR5*  a. 57% in K562 cells  *AAVS1*  a. 72% in K562 cells  iii. RNPs is more effective than liposome  iv. Cas9 from *E.coli*  v. Off-target reduced |
| Schwank (2013)  5^th^ TC2020 | Editing system to correct the CF transmembrane receptor locus | a. Organoids derived from cystic fibrosis (CF) patients | CRISPR-Cas9 with single gRNA  Liposome | i. In vitro research  ii. Gene edit  *CFTR*  a. 1.6% knockout mutation  b. 0.3% knockin mutation  iii. 60-80% increased surface area  iv. Off-target occur but no indels |
| Koike-Yusa (2014)  6^th^ TC2020 | Knockout screening for mouse genes | Mouse embryonic stem cells | CRISPR-Cas9 with single gRNA  Lentivector | i. In vitro research  ii. Gene edit  *PIGA*  a. 12.7 ± 6.7 % indel frequency  iii. Indel frequency caused the gene produced resistant cells against *Clostridium septicum* alpha-toxin or 6-thioguanine  iv. Replaced the first nucleotide in U6 promoter with a G for sites 1 and 3 to increase the expression |
| Tabebordbar (2016)  7^th^ TC2020 | Edit gene that causes dystrophic mouse muscle and muscle stem cells | Mouse muscle cells | CRISPR-SaCas9 with paired gRNA  Adenovector | i. In vivo research  ii. Gene edit  *DMD*  a. 24%-47% causes deletion in dystrophic satellite cells  b. 3%-18% in adult dystrophic muscle cells |
| Lin (2014)  8^th^ TC2020 | Introduce HDR-CRISPR system that can edit human genome | a. HEK293T  b. human primary neonatal fibroblast  c. human embryonic stem cells | CRISPR-Cas9 with single gRNA  Nucleofection-RNP complex | i. In vitro research  ii. Gene edit  *CXCR4*  a. 38% knockin in HEK293T cells  b. 50% knockout in HEK293T cells  *EMX1*  a. 17% knockout in human primary neonatal cells  b. 1.3% knockin in human primary neonatal cells  c. 48% knockout in human embryonic stem cells  d. 1.6% knockin in human embryonic stem cells  iii. Addition of nocodaloze enhance CRISPR performance |
| Wu (2014) 9^th^ TC2020 | Mapping CRISPR/Cas9 binding sites in mammalian | Mouse embryonic stem cells (mESCs) | CRISPR/Cas9 with single gRNA with lipofectamine transfection (liposome) | i. In vitro research  ii. Gene edit  a.*Phc1* promoter  12-37% indel in in mouse embryonic stem cells (mESCs)  b. *Nanog* promoter  70% off-target in mESCs because of chromatic inaccessibility  iii. Active genes were easier to be disrupted compared to enhancer or promoter. |
| Matano (2015) 10^th^ TC2020 | Developing colorectal cancer model for organoid | Human intestinal organoid | CRISPR/Cas9 with plasmid electrophoration | i. In vitro research  ii. Gene edit  a. 0.04% *TP53* gene knockout in adenoma intestinal organoid  b. 0.03% *APC* gene knockout in normal intestinal organoid |
